# Predicting antimicrobial resistance in *E. coli* with discriminative position fused deep learning classifier

**DOI:** 10.1016/j.csbj.2023.12.041

**Published:** 2023-12-29

**Authors:** Canghong Jin, Chenghao Jia, Wenkang Hu, Haidong Xu, Yanyi Shen, Min Yue

**Affiliations:** a School of Computer and Computing Science, Hangzhou City University, Hangzhou 310015, China; b Institute of Preventive Veterinary Sciences and Department of Veterinary Medicine, Zhejiang University College of Animal Sciences, Hangzhou 310058, China; c Hainan Institute of Zhejiang University, Sanya 572000, China; d Zhejiang Provincial Key Laboratory of Preventive Veterinary Medicine, Hangzhou 310058, China; e State Key Laboratory for Diagnosis and Treatment of Infectious Diseases, National Clinical Research Center for Infectious Diseases, National Medical Center for Infectious Diseases, The First Affiliated Hospital, College of Medicine, Zhejiang University, Hangzhou 310003, China; f College of Computer Science and Technology, Zhejiang University, Hangzhou 310027, China

**Keywords:** Antimicrobial resistance, Machine Learning, *E. coli*, Feature Augmentation, Whole-Genome Sequencing, Single Nucleotide Polymorphism

## Abstract

*Escherichia coli* (*E. coli*) has become a particular concern due to the increasing incidence of antimicrobial resistance (AMR) observed worldwide. Using machine learning (ML) to predict *E. coli* AMR is a more efficient method than traditional laboratory testing. However, further improvement in the predictive performance of existing models remains challenging. In this study, we collected 1937 high-quality whole genome sequencing (WGS) data from public databases with an antimicrobial resistance phenotype and modified the existing workflow by adding an attention mechanism to enable the modified workflow to focus more on core single nucleotide polymorphisms (SNPs) that may significantly lead to the development of AMR in *E. coli.* While comparing the model performance before and after adding the attention mechanism, we also performed a cross-comparison among the published models using random forest (RF), support vector machine (SVM), logistic regression (LR), and convolutional neural network (CNN). Our study demonstrates that the discriminative positional colors of Chaos Game Representation (CGR) images can selectively influence and highlight genome regions without prior knowledge, enhancing prediction accuracy. Furthermore, we developed an online tool (https://github.com/tjiaa/E.coli-ML/tree/main) for assisting clinicians in the rapid prediction of the AMR phenotype of *E. coli* and accelerating clinical decision-making.

## Graphical Abstract

**Figure:**
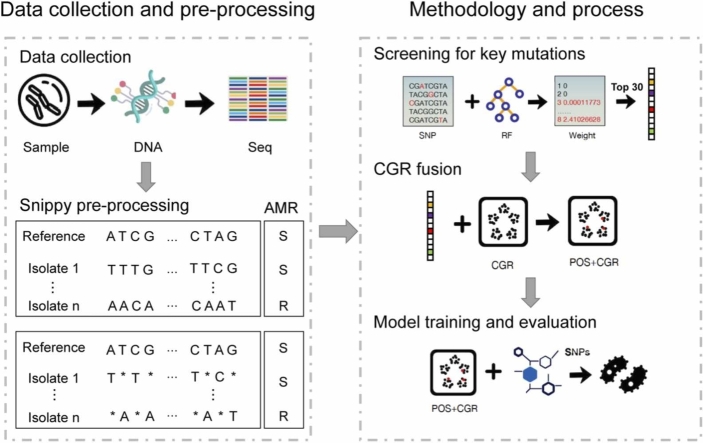

## 1 Introduction

Antimicrobial resistance (AMR) has become a major public health challenge of the 21st century [1], [2], and an estimated 10 million people could be killed per year by 2050 [3]. *E. coli*, as a major bacterial pathogen in clinical settings and frequently resistant to multiple antimicrobials, is a significant concern across many countries. Typically, antimicrobial susceptibility testing relies on culture-based methods and presents minimum inhibitory concentration (MIC) values [4], [5], [6], which are time-consuming and difficult to manipulate. Genetic approaches were a potential candidate to replace traditional methods. However, detection relies on measuring a reference set of genes, which requires prior knowledge of their biological mechanisms [7], [8], [9], [10]. To address this problem, machine learning (ML) has been developed in which the algorithm takes input examples labelled for a particular output and trains until it can recognize the underlying patterns and relationships between the input data and the output labels, enabling it to produce accurate results and overcome the limitations of rule-based testing [11].

Single nucleotide polymorphisms (SNPs) are variations in the DNA sequence of single nucleotides that can cause *E. coli* to become resistant to multiple types of antimicrobials [12], [13], [14]. As an unbiased input, SNPs sets have been successfully used for predicting bacterial AMR in previous studies, with two categories of ML methods: feature-based methods [15], [16] and image-based methods [17], [18]. Feature-based methods are generally based on one-hot encoding [19] and label encoding [20]. In one-hot encoding, each amino acid is represented as a one-hot vector, and in label encoding, each label is a unique integer. Conversely, image-based methods transfer SNPs into related images and then use image classification models to predict AMR results. Among them, Chaos Game Representation (CGR) [21] is a holistic approach that considers SNP sets as strings composed of different units and obtains images after constant rule transformation.

Although deep convolutional neural networks (CNN) outperform [22] feature-based models in most image classification tasks, it is difficult to determine which part of the image plays a more significant role in prediction. The attention mechanism [23] is inspired by the human biological system, which tends to focus on distinct parts when dealing with large amounts of information. Neuro-attentional mechanisms can endow neural networks with the ability to focus on a subset of their features and select specific inputs, which can significantly improve the performance of the CNN [24]. Here, we developed a novel, high-performance workflow and demonstrated that the prediction performance can be enhanced by integrating discriminative positional features selected by random forest (RF) model in CGR-related images. Additionally, we systematically evaluated the performance of various ML methods for predicting antimicrobial resistance from *E. coli* core SNPs sets.

## 2 Materials and methods

### 2.1 Data preparation

A total of 1937 *E. coli* genome sequences were retrieved from the European Nucleotide Archive. Six categories and 12 classes of antimicrobial resistance phenotype profiles were collected: aminoglycosides (tobramycin TBM, gentamicin GEN), quinolone (ciprofloxacin CIP), beta-lactams (amoxicillin AMC, thiazolopyrimidine TZP), cephalosporins (cefuroxime CXM, cetirizine CET, ceftazidime CTX, ceftazidime CTZ), sulfonamide (trimethoprim TMP), and penicillin (ampicillin AMP, amoxicillin AMX). Notably, not all isolates have a complete antimicrobial resistance phenotype profile, the antimicrobials commonly used in clinical practice [25] (GEN, CIP, CXM, CTX and CTZ) have the most comprehensive information. The corresponding antimicrobial resistance phenotype profiles were previously categorized into three groups based on antimicrobial resistance levels: resistant (R), susceptible (S), and intermediate (I) (Supplementary Table 1). In this study, we labelled neutral intermediate strains as "S" and divided the entire database into three parts, which were used for training (80%), validation (10%), and testing (10%), respectively.

### 2.2 Database descriptions

For antimicrobial susceptible and resistant isolates, we observed a particular distribution bias, with antimicrobial susceptible isolates greatly outnumbering antimicrobial resistant isolates (Fig. S1A). Specifically, among the 1935 *E. coli* tested for TZP susceptibility, 27% were resistant, while the remaining 73% were susceptible. The ratio of resistant to susceptible isolates was approximately 1:15.6. This ratio was also skewed among other antimicrobials showing a similar pattern, such as CXM, CIP, GEN, CTZ, and CTX, with the ratos being 1:2.7, 1:3.5, 1:5.8, 1:6.3, and 1:2.9, respectively.

### 2.3 Pre-processing and calculation of core SNPs

SNIPPY (Ver 3.1.0) was utilized to identify the core single nucleotide polymorphisms (core SNPs) between the *E. coli* genome sequence and the reference gene (GCF_904425475.1) with specific parameter configurations: the number of processing units (ncpus) was set to 16, the minimum read mapping quality (mapqual) was established at 80, the minimum site depth for calling alleles (mincov) was defined as 12, and the minimum QUALITY in the VCF column (minqual) was set to 100. All other parameters were maintained at their default settings. The recombinant regions were removed using Gubbins [26]. Next, the identified core SNPs sequences of each isolate were obtained using a local Python3 script and merged according to the position of the reference alleles. The " core SNPs " were defined as single nucleotide polymorphisms that occur in each whole-genome sequence data. We left the mutated alleles unchanged and replaced the unmutated alleles with “*”, and constructed a core SNPs matrix where the rows represented the samples, and the columns represented the mutated nucleobases at different positions. The average length of core SNPs is approximately 25,000–35,000 bp for distinct isolates (Fig. S1B).

### 2.4 Core SNPs matrix encoding

Label encoding and CGR encoding were used to convert the core SNPs matrix into an input-ready data format. For label encoding, “A”, “G”, “C”, “T”, and “*” in the core SNPs matrix are converted to 0, 1, 2, 3, and 4. For the CGR encoding and the FCGR encoding, we converted the core SNPs matrix into a two-dimensional image representation using the proposed methods [21], [27], with the resolution set to 200.

### 2.5 Feature selection and integration

The lengths of core SNPs among antimicrobial susceptible and resistant strains exhibit considerable variation. To enhance the performance and reduce the computational complexity of the modeling, we employ a feature selection method (ATT+POS). This method assists the ATT method by allowing it to focus more on the filtered features. The selected features were considered to be the most informative and relevant for predicting susceptibility or resistance. The selection RF method was utilized to assign weights to the features of each core SNPs position, which used bootstrap aggregation to reduce the variance of each subtree. The Gini index was used to filter the main characteristics of the RF model and was calculated as follows, where pi is the probability that a feature belongs to category “I”.

$${\mathrm{Gini}(s}_{t})=1-\sum_{i=1}^{C}p_{i}^{2}$$

The importance weights of each feature were ranked in descending order, and the top 30 core SNPs positions were identified as critical mutation sites. We mapped all Top30 features onto an image C of the specified resolution as follows:

$$C[T_{m}]=C[T_{m}]+p_{m}\times \in$$

Where p_m_ is the importance measure of the feature point, T_m_ represents the pixel position of the m-th nucleotide in the gene sequence within the image, and ε is the importance adjustment factor. We also emphasize and display (highlight colors) those critical mutation positions to increase additional attention to these key locations during the image recognition process (Fig. S2).

### 2.6 Construction of the model and parameters setting

We used distinct traditional ML methods, including logistic regression(LR), support vector machine [5], random forest [28], to forecast the susceptibility of *E. coli* to specific antimicrobials. All procedures were implemented using the scikit-learn [29] package. The parameters encompass patch size set to 14, a batch size of 32, 50 epochs, the Adam optimizer, and a learning rate of 1e-5. Additionally, GridSearchCV and KFold (k-fold cross-validation) were used to identify the optimal combination of hyperparameters. The corresponding search values are presented in Table 1. Parameters with a singular value are explicitly defined, and those with multiple values undergo optimization through a search process.

**Table 1:** The LR, RF, and SVM parameters consist of multiple values.

ML Methods	param	value
LR	C	0.5,1,2
RF	n_estimators	50, 100, 200
	max_depth	none, 10, 20
	min_samples_split	2, 5, 10
	min_samples_leaf	1, 2, 4
	bootstrap	true, false
SVM	C	0.1, 1, 10, 100
	kernel	'linear', 'rbf', 'poly', 'sigmoid'
	gamma	‘scale’, ‘auto’

**Table 2:** Encoding methods employed for each model.

Model	Encoding Methods
RM	Label Encoding
LR	Label Encoding
SVM	Label Encoding
CNN	CGR
ATT	CGR
ATT+POS	CGR+POS

Convolutional Neural Network (CNN) models were employed, including a Keras-based non-attention mechanism CNN model and a TensorFlow-based attention mechanism CNN model (ATT, ATT+POS). The input comprised 224 × 224 × 3 images, which were initially divided into 8 × 8 patches and then transformed into one-dimensional sequences. The transformer module comprised eight layers, each combining the attention and feedforward modules, with a batch size of 32, and the training consists of 50 epochs. The number of heads in the attention module was 4, and the dimension of the hidden layer was 64. The final output was passed through the flattened and linear layers to obtain a logical output. Both the CNN and ATT methods utilized the Adam [30] optimizer with a learning rate of 0.0002, while the ATT method further incorporated a weight decay of 0.0001. Furthermore, both methods employed Dropout [31] and Batch LayerNormalization [32] for mitigating overfitting.

### 2.7 Model evaluation

The performance of feature-based ML and deep learning models was evaluated in terms of precision, recall, F1-score, MCC, AUROC, and AUPRC, which are defined as follows:

(1) $$\mathrm{Precision}=\frac{\mathrm{TP}}{\mathrm{TP}+\mathrm{FP}}$$

(2) $$\mathrm{Recall}=\frac{\mathrm{TP}}{\mathrm{TP}+\mathrm{TN}}$$

(3) $$F_{1}-\mathrm{Score}=\frac{2\times \mathrm{TP}}{2\times \mathrm{TP}+\mathrm{FP}+\mathrm{FN}}$$

(4) $$\mathrm{MCC}=\frac{\mathrm{TP}\times \mathrm{TN}-\mathrm{FP}\times \mathrm{FN}}{\sqrt{\left(\mathrm{TP}+\mathrm{FP})(\mathrm{TP}+\mathrm{FN})(\mathrm{TN}+\mathrm{FP})(\mathrm{TN}+\mathrm{FN}\right)}}\;$$

Where TP was the number of resistant strains predicted to be resistant, TN was the number of sensitive strains predicted to be sensitive, FP was the number of sensitive strains predicted to be resistant, and FN was the number of resistant strains predicted to be sensitive.

(5) $$\mathrm{AUROC}=\int_{0}^{1}\mathrm{TPR}\left(\mathrm{FP}R^{-1}\left(t\right)\right)\mathrm{dt}$$

Where TPR and FPR are defined as follows:

(5.1) TPR=TP/(TP+FN)

(5.2) FPR=FP/(FP+TN)

$\mathrm{AUPRC}=\int_{0}^{1}p\left(r\right)\mathrm{dr}$

**Fig. 1:**
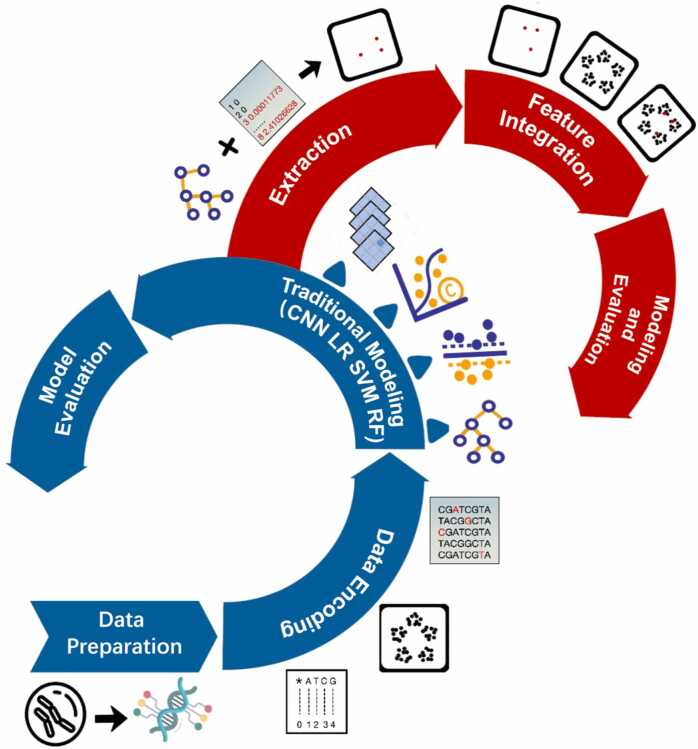
Workflow of this study. Whole genome sequences of *E. coli* were collected from the European Nucleotide Archive, and core SNPs were processed by SNIPPY software. The core SNPs were encoded by two different methods: CGR and label. Four standard models (CNN, LR, RF, SVM) and two CNN algorithms that include an attention mechanism (ATT, ATT+POS) were employed individually. For ATT+POS, we used Sk-learn software with random forest algorithms to select the discriminating position for each core SNPs (top 30). Finally, the six models were assessed using various metrics, including precision, recall, F1-score, MCC, AUROC, and AUPRC.

The whole workflow used in this study is depicted in Fig. 1.

## 3 Results

### 3.1 Attention regions estimated by improved workflow

**Fig. 2:**
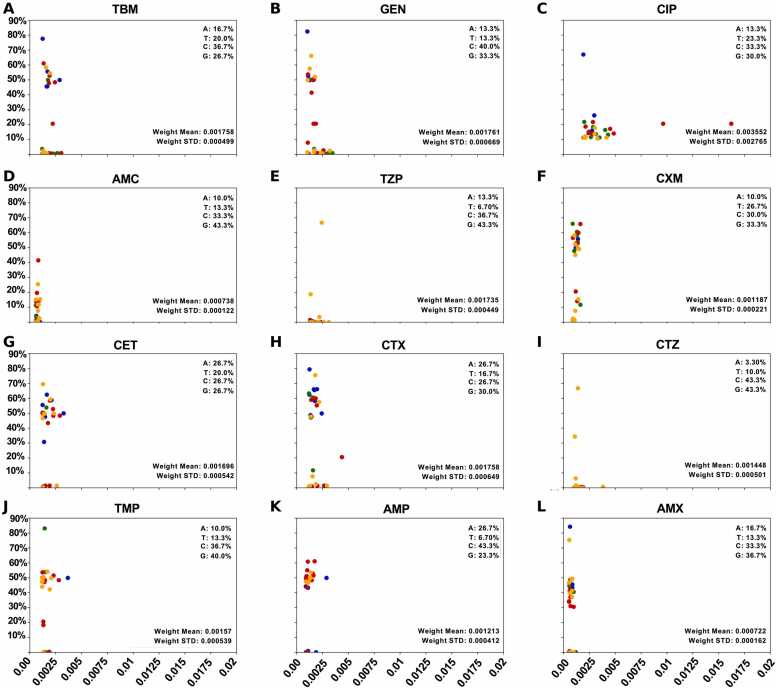
The mutation frequency of core SNPs with the Top30 weight within each antimicrobial categories. Different colours represent four different types of nucleobases (A, T, C and G). The proportion of each nucleobase type and the mean/standard deviation of these weighted scores are shown below.

Using the RF algorithm, the core SNPs with the top 30 weights were selected for each antimicrobial **(**Fig. 2). Our findings indicate that the combination of nucleobase types "G" and "C" exhibited more substantial weight than the combination of nucleobase types "A" and "T". Particularly for antimicrobials such as TMP, AMX, CTZ, and GEN. However, the overall weight of the top 30 core SNPs sets was similar for different antimicrobials. The highest and lowest weights of the top 30 core SNPs were for CIP (0.0035) and AMX (0.0007), respectively.

**Fig. 3:**
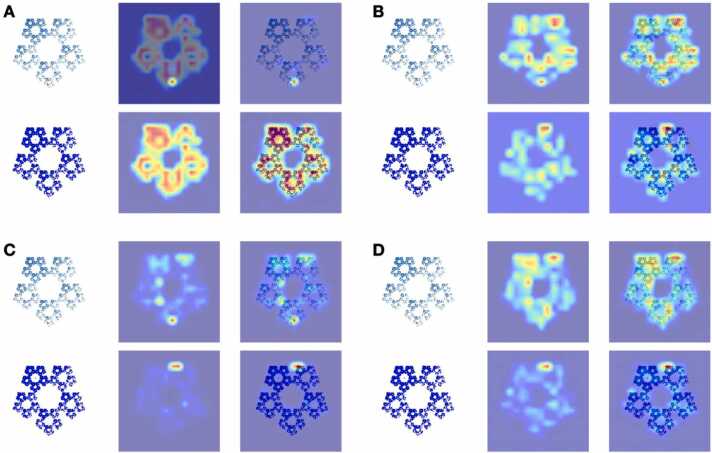
The CGR image of *E. coli’* core SNPs sequence. A, B, C, and D represent the four heads in the multi-head attention mechanism. The light color in the first column represents the CGR image without any prior positional information. The second column represents the CGR image incorporating attentional positional information and the third column represents the superposition of the first column and second column.

To evaluate the effect of the attention heatmap on CGR images, *E. coli’* top 30 core SNPs sequences were selected and compared before and after incorporation with the CGR images (Fig. 3). The CGR images of isolates exhibited a substantial increase in network density and concentration after the integration of the attention heatmap. Prior to the heatmap, the network was usually dispersed. Consequently, our findings indicate that the attention heatmap enables the neural network to allocate more processing resources to critical components by directing attention to weighted portions.

### 3.2 Cross-performance of phenotype prediction machine learning algorithms

Ten percent of the strains from the dataset were utilised as a testing dataset. To assess the cross-performance of various classifiers on the testing dataset, we present a comparative analysis of each model's performance using multiple evaluation metrics, including “Precision”, “Recall”, “F1 score”, “MCC”, “AUROC”, and “AUPRC”, (Supplementary Table 2). The selected evaluation metrics indicate the quality of the model's predictions, with higher scores corresponding to better performance.

Considering the number of antimicrobial-sensitive strains exceeds the number of antimicrobial-resistant strains in the training dataset, we primarily consider the AUROC and AUPRC metrics to evaluate the ML methods as they address the potential unbalance of the dataset. Additionally, we employ the F1-Score and MCC as supplementary evaluation criteria. Overall, we observed a slight variation in performance between the models using LR, RF and SVM algorithms. Among them, RF showed better predictive performance for antimicrobial resistance phenotypes of *E. coli*. Specifically, using the RF method, we observed that the average AUROC for 12 antimicrobials was 0.72, F1-score was 0.68, while AUPRC and MCC had mean values of 0.49 and 0.42, respectively. Notably, the RF method exhibits superior performance for specific antimicrobials such as ciprofloxacin, cetirizine, and ceftazidime.

**Fig. 4:**
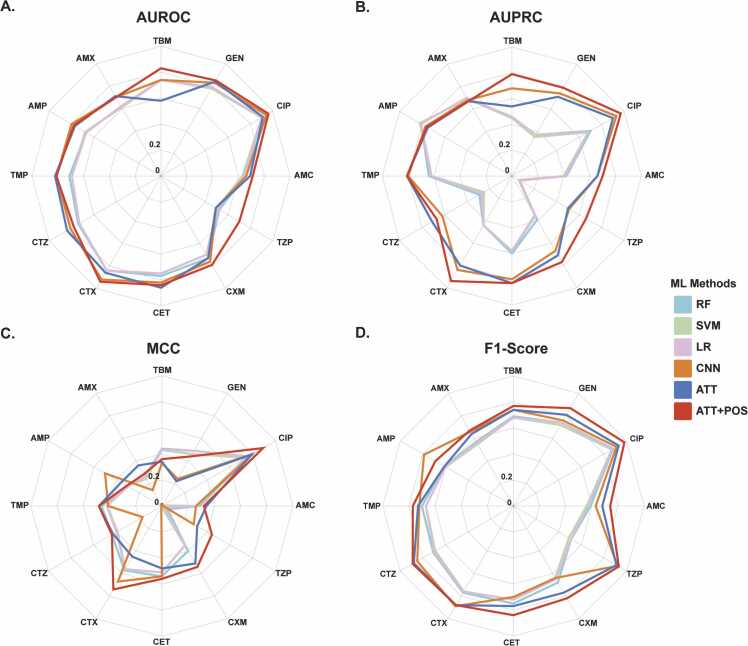
Machine learning methods are assessed using four key criteria: AUROC, AUPRC, MCC, and F1-Score. These metrics will be denoted as A, B, C, and D, respectively. Distinct line colors are used to represent each ML method.

On the contrary, deep learning models exhibit superior performance compared with traditional machine learning models. The results demonstrate that the attention mechanism improved the accuracy of the CNN model in predicting antimicrobial resistance phenotypes in *E. coli*. The CNN model achieved a mean AUROC of 0.77 and an F1 score of 0.77 for 12 antimicrobials. Additionally, the mean values of AUPRC and MCC were 0.725 and 0.36, respectively. Upon adding the attention mechanism, the model's F1-score and MCC increased to 0.78 and 0.42, respectively, while the AUROC and AUPRC scores decreased. Remarkably, the improved model with attention mechanism and POS (ATT+POS) displayed significant enhancements in AUROC, AUPRC, MCC, and F1-Score. In comparison to the CNN method, the average AUROC improved to 0.8, the average AUPRC improved to 0.78, while the average MCC and F1-score improved to 0.48 and 0.82, respectively. These results suggest that incorporating POS information in the CGR image can significantly enhance the performance of the ATT model and provide a greater performance boost to the CNN (Fig. 4).

## 4 Discussion

In this study, we propose a new model that directly combines discriminative features with attentional mechanisms without priori knowledge (ATT+POS). The results demonstrate that the deep learning model with an attention mechanism significantly outperforms the prediction results for seven of the 12 available antimicrobials. Our study also shows that discriminative positional colours in CGR images could influence and highlight the regions of interest, thus improving the classification accuracy of core SNPs data.

Machine learning models have been applied to antimicrobial resistance analysis in previous studies. For example, the self-attention layer model can learn convolutional filters. The crucial difference between transformer models and traditional neural network modules, such as CNNs and recurrent neural networks, is that transformer models focus on each position of their input sequence simultaneously through an attention mechanism.

Specifically, we show that a multi-headed attention layer achieves expressive power beyond that of CNNs when the number of heads is sufficiently large. In fact, the multi-headed attention layer covers the convolutional layer in terms of expressive power. Instead of learning features on a fixed grid, it recognizes the location of the receptive areas over the entire image. The receptive field of self-attention is always a complete image in a neighbourhood grid. Attention probability is generally determined by first calculating the weights of each value in the sequence based on the input query and then normalizing them. The input vector in the attention mechanism often adds positional information by adding positional encoding (via addition or concatenation). To apply the attention mechanism to an image, position encoding is first learned for each pixel in the image and then added to the representation of the image itself. Moreover, because of the multi-head attention mechanism, each head can focus on a different part of the image (position or content) for each query pixel.

However, there are also some limitations to our study that should be considered. Biologically, the core SNPs loci with the highest weights chosen by the random forest algorithm are arbitrary. While there might exist a correlation between the location and type of these mutations and the emergence of antimicrobial resistance in *E. coli*, the precise mechanism requires further investigation. Secondly, in biological experiments for determining *E. coli* resistance, a specific MIC value is often provided, but current models only predict resistance or non-resistance. This limitation hinders obtaining more comprehensive drug resistance information for isolates. Finally, from an algorithmic perspective, the model-building process often generates a large number of parameters. Understanding the biological significance of these parameters is challenging. For instance, we observed that the degree of performance improvement varies depending on the antimicrobial. Integrating an attention mechanism into the CNN models can lead to a notably greater improvement for aminoglycosides, quinolones, beta-lactamases, and cephalosporins. Therefore, further investigation is warranted to provide a more rational explanation for this difference in performance.

## Author statement

Canghong Jin and Min Yue designed the study; finalized the manuscript; Chenghao Jia and Wenkang Hu collected isolates and clinical data, integrated the data, performed metadata and genomic analysis, prepared tables and figures, and wrote the draft manuscript; Haidong Xu performed genomic analysis; interpreted the data; Yanyi Shen prepared figures; M.Y. managed the project; All the authors contributed to the article and approved the submitted version.

## Declaration of Competing Interest

The authors declare that they have no known competing financial interests or personal relationships that could have appeared to influence the work reported in this paper.

## Data Availability

The datasets used in this study are open access. The raw sequences of the genomic data from the European Nucleotide Archive (ENA) are available at: https://www.ebi.ac.uk/ena/browser/view/PRJEB45963. The reference strain used for the Snippy comparative analysis is available at: https://www.ncbi.nlm.nih.gov/assembly/GCF000005845.2. The online tool for assisting clinicians in the rapid prediction of AMR of *E. coli* and accelerating clinical decision-making is available at: https://github.com/tjiaa/E.coli-ML/tree/main.
