# Supplementary material for: Predicting antimicrobial resistance in E. coli with discriminative position fused deep learning classifier
Source: Comput Struct Biotechnol J. 2023 Dec 29;23:559–65. doi: 10.1016/j.csbj.2023.12.041 (PMC10809114; doi:10.1016/j.csbj.2023.12.041)
Supplement: Supplementary file 1 — Supplementary material [file mmc1.docx]

**Supplementary Figures**

**Figure S1.** The number of sensitive/resistant strains and the distribution of core SNPs sequence length within the different antimicrobial categories. (A) The stack diagram shows the counts of susceptible and resistant strains of each antimicrobial. (B) The bar shows the mean and standard deviation of the length of core SNPs sequences between susceptible and resistant strains within different antimicrobial categories.

**Figure S2.** The RF method filters the Top30 features, which are then represented in Chaos Game Representation (CGR) images.

**Supplementary Tables**

**Table S1.** Summary of 1,937 *E. coli* genome sequences retrieved from the European Nucleotide Archive and corresponding antimicrobial resistance phenotype information.

**Table S2.** Different ML methods’ performance for various categories of antimicrobials.

**Table S3.** Download link for 1,937 *E. coli* genome sequences.

**Table S3.** Specific parameters for each layer of the CNN, including the number of kernels and kernel sizes.
