# Supplementary figures and images for: Predicting antimicrobial resistance in E. coli with discriminative position fused deep learning classifier
Source: Comput Struct Biotechnol J. 2023 Dec 29;23:559–65. doi: 10.1016/j.csbj.2023.12.041 (PMC10809114; doi:10.1016/j.csbj.2023.12.041)

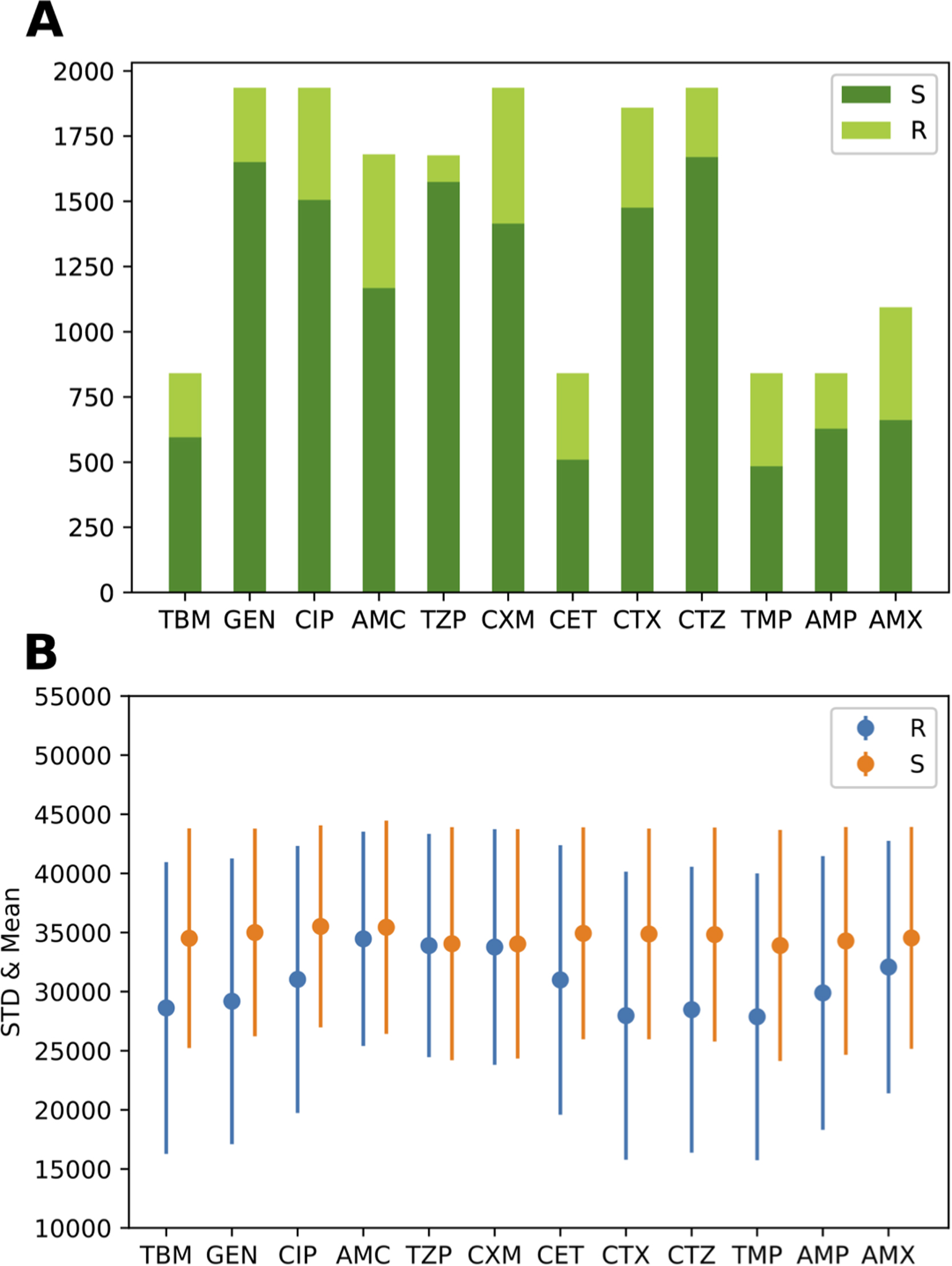

Supplement: Supplementary file 6 — Supplementary material [file mmc6.jpg]

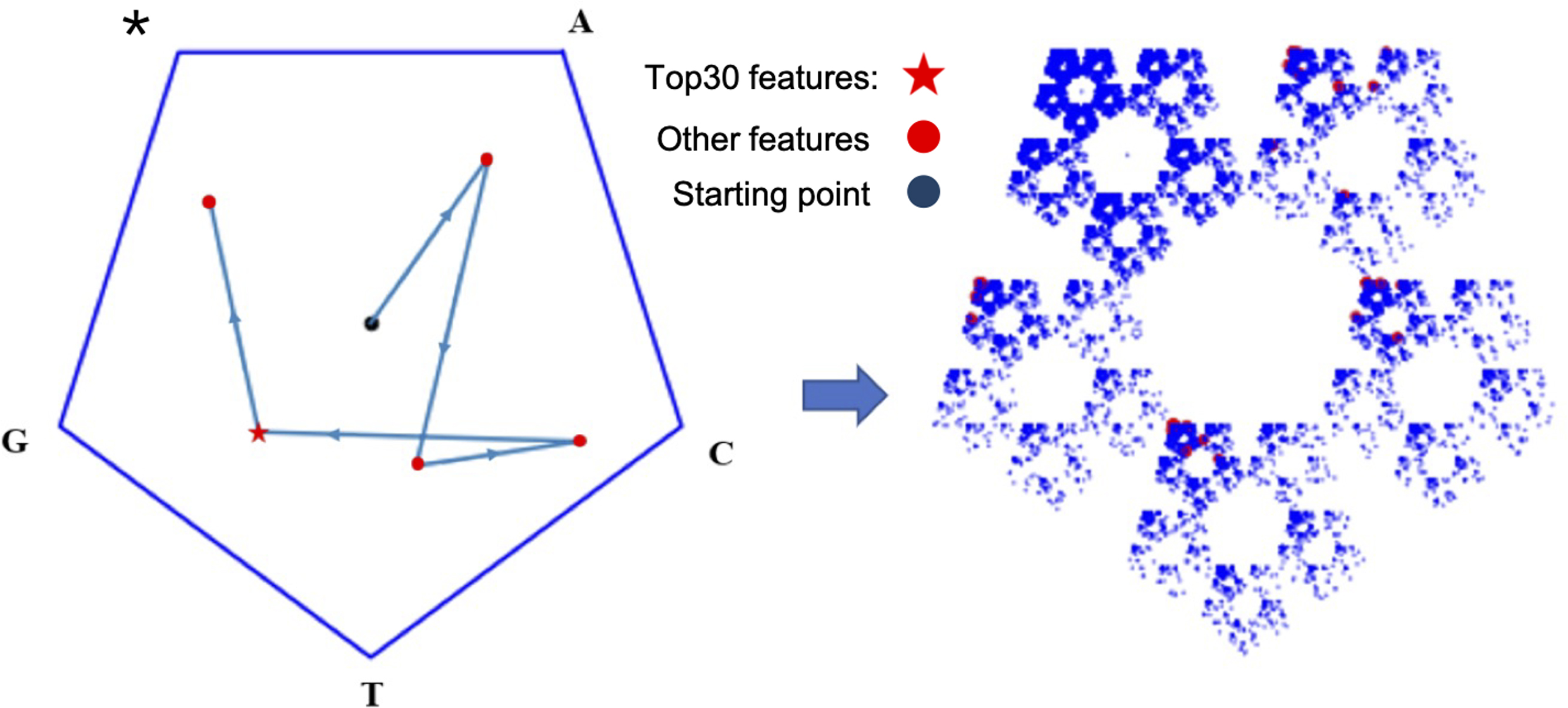

Supplement: Supplementary file 7 — Supplementary material [file mmc7.jpg]
